# Who uses Australian chiropractic services?

**DOI:** 10.1186/2045-709X-21-31

**Published:** 2013-09-26

**Authors:** Simon D French, Konstancja Densley, Melanie J Charity, Jane Gunn

**Affiliations:** 1 General Practice and Primary Health Care Academic Centre, The University of Melbourne, 200 Berkeley Street, Carlton, VIC 3072, Australia; 2 Centre for Health, Exercise and Sports Medicine, School of Health Sciences, The University of Melbourne, Level 7, 161 Barry St, Carlton, VIC 3053, Australia

## Abstract

**Background:**

The use of chiropractic services is widespread, however, little is known about the characteristics of people who seek chiropractic care in Australia. This study compared the characteristics of users and non-users of chiropractic services from a cohort of patients sourced from general medical practice in Victoria, Australia.

**Methods:**

This is a secondary analysis of baseline screening data from a prospective adult cohort study beginning in 2005. Thirty randomly selected Australian general medical practices mailed out surveys to 17,780 of their patients. Differences were examined between chiropractic users and others, and between chiropractic users who reported a back problem to those who did not.

**Results:**

Of 7,519 respondents, 15% indicated they had visited a chiropractor in the last 12 months. Chiropractic users were more likely to have their GP located in a rural location and to be born in Australia; they were less likely to be in the older age group (55–76), to be unemployed or to have a pension/benefit as their main source of income. Chiropractic users were more likely to: have a back problem; use complementary or alternative medication; visit another type of complementary health practitioner or a physiotherapist. They were less likely to take medication for certain health problems (e.g. for high blood pressure, high cholesterol or asthma). No important differences were seen between chiropractic users and non-users for other health problems. People who visited a chiropractor and reported a back problem were more likely to: be a current smoker; have a number of other chronic conditions, including arthritis, hypertension, chronic sinusitis, asthma, dermatitis, depression and anxiety; report taking medications, including antidepressants, analgesics (painkillers and arthritis medication) and complementary or alternative medications.

**Conclusions:**

This large cross-sectional study of general medical practice attendees suggests that chiropractors are the most commonly consulted complementary health profession. Chiropractors should ensure they are aware of their patients’ health conditions other than musculoskeletal problems and should ensure they are appropriately managed.

## Background

The use of complementary and alternative medicine is widespread across most high income countries [1]. Chiropractic services account for a substantial proportion of this use. There are approximately 4,500 registered chiropractors in Australia, the ninth largest registered health profession (out of 12) [2]. During 2005, 16% of Australians consulted a chiropractor at least once at a cost of $AU905 million [3]. Further, in 2011, Australian private insurers paid approximately AUD$225 million for more than 9 million chiropractic services, almost as much as was paid for physiotherapy services (AUD$258 million) [4].

Most previous studies of chiropractic users have been conducted in the United States and European countries [5-9]. These studies included patients from chiropractic practices rather than from the general population.

Despite a high level of use of chiropractic in Australia, little is known about the characteristics of people who use chiropractic care. Two previous Australian population-based studies about people who consult chiropractors found that chiropractic users, compared to non-users, have a higher income, were more likely to be born in Australia, and were more likely to have also visited a general medical practitioner [3,10]. A study of chiropractic patients in Victoria, Australia, demonstrated that although chiropractors see patients with a range of conditions, most commonly these conditions are musculoskeletal-related [11].

This paper offers a view on how current users of general medical practice use chiropractic services. For general practitioners, it is important to know which of their patients use chiropractic services; more than half of people who use complementary and alternative services and treatments do not reveal this to their general practitioner [12]. For chiropractors, this analysis can reveal important characteristics of the patients who seek their care. The aim of this study was to compare the characteristics of users and non-users of chiropractic services from a cohort of patients sourced from general medical practice. For those people who saw a chiropractor, we also compared the characteristics of those who reported a back problem to those who did not.

## Methods

We undertook a secondary analysis of screening data from *diamond*. The *diamond* study began in 2005 and was large prospective study exploring depression, stress and worries in people presenting to primary care. Full details of the study are reported elsewhere [13,14], and the methods are briefly outlined below. Ethics approval for the *diamond* study was granted by the University of Melbourne’s Human Research Ethics Committee.

### Participant sample

Thirty randomly selected general practices located in Victoria, Australia, mailed out surveys to randomly selected patients. Patients were eligible if they were: aged 18–75 years; able to read English; not terminally ill; and did not reside in a nursing home.

### Measures

The survey included questions on general demographic information, general health, medication use and health service use in the previous 12 months. Participants were asked whether they had seen any traditional health professionals (hospital doctor, specialist doctor, physiotherapist, psychologist, counsellor, psychiatrist, nurse, social worker, alcohol or drug worker or family therapist) or a complementary therapist (chiropractor, naturopath, homeopath, acupuncturist or other natural therapist) in the previous 12 months.

### Statistical analysis

Data were analysed using Stata version 12 [15] and summarised using frequencies and percentages. Participants were divided into two groups according to whether they had consulted a chiropractor or not in the past 12 months. Logistic regression using generalised estimating equations with robust standard errors was used to examine the demographic characteristics, health issues, and medications taken of people who had consulted a chiropractor and to investigate the association between visiting a chiropractor and other health service use. A further two groups were formed consisting of those who had a back problem, chronic back pain or sciatica and saw a chiropractor, compared to those who saw a chiropractor and didn’t have a back problem.

Analyses allowed for the clustering effect due to recruiting participants from the same general practices. Results are reported as odds ratios (ORs) with 95% confidence intervals (CI) and p values (P). Analyses investigating the association between visiting a chiropractor and other health service use were controlled for the effects of age, sex, general practice location and health rating.

## Results

Of the 17,780 patients initially sent a screening survey, 7,667 (43%) returned a completed survey. The mean age of patients who were sent the screening survey was 46.2 years (SD, 15.3) and 61% were women. Patients who returned the survey were on average older (50.9 years; SD, 14.2) and more likely to be female (67%).

A total 7,519 participants answered the question on whether they had consulted a chiropractor in the past 12 months and of those, 7,477 people responded to the question related to back problem, chronic back pain or sciatica. Fifteen per cent of respondents (N = 1,134) indicated they had visited a chiropractor in the last 12 months. Chiropractors were the fifth most common health professional visited after specialist doctor (47%), nurse (29%), hospital doctor (21%) and physiotherapist (21%) and the most commonly visited complementary health professional.

Important differences in demographic characteristics between people who reported they had consulted a chiropractor in the last 12 months, compared to people who didn’t, included that they were more likely to have their GP located in a rural location (Odds Ratio (OR) 1.29, 95% Confidence Intervals (CI) 1.03, 1.62) and to be born in Australia rather than another country (OR 1.44, 95% CI 1.21, 1.72). They were less likely to be in the age group of 55–76 (OR 0.73, 95% CI 0.62, 0.87), to have a pension or benefit as their main source of income (OR 0.58, 95% CI 0.47, 0.71) or to be unemployed (OR 0.69, 95% CI 0.59, 0.82) (Table 1).

**Table 1:** Participant demographic characteristics (N = 7,519)

	**Visit to chiropractor in last 12 months**^**1**^					
	**No (N = 6385)**		**Yes (N = 1134)**			
**Participant characteristics**	**Number**	***%***	**Number**	***%***	**OR (95% CI)**^**2**^	**P**
General practitioner location^**3**^:						
- Urban (RRMA 1 and 2)	4363	*68*	711	*63*	REF	0.03
- Rural (RRMA 3 to 5)	2022	*32*	423	*37*	1.29 (1.03 – 1.62)	
Age group:						
18-34	974	*15*	185	*16*	REF	<0.001
35-54	2631	*42*	562	*50*	1.11 (0.92 – 1.35)	
55-76	2712	*43*	378	*34*	0.73 (0.62 – 0.87)	
Gender: Female	4231	*67*	757	*67*	1.02 (0.87 – 1.20)	0.81
Marital status:						
- Never married/single	1139	*18*	202	*18*	REF	0.24
- Widowed/divorced/separated	1149	*18*	179	*16*	0.88 (0.74 – 1.06)	
- Married	4027	*64*	741	*66*	1.03 (0.87 – 1.23)	
Born in Australia	5104	*80*	972	*86*	1.44 (1.21 – 1.72)	<0.001
English is first language	6012	*95*	1087	*96*	1.24 (0.88 – 1.75)	0.22
Lives alone	877	*14*	130	*12*	0.82 (0.69 – 0.97)	0.02
Highest level of education:						
- Completed year 12 or less	3605	*57*	618	*55*	REF	0.07
- Certificate/diploma	1292	*20*	260	*23*	1.19 (1.02 – 1.38)	
- Bachelor degree or higher	1448	*23*	254	*22*	1.04 (0.89 – 1.21)	
Employment:						
- Employed/student	3970	*62*	815	*72*	REF	<0.001
- Not employed	2018	*32*	286	*25*	0.69 (0.59 – 0.82)	
- Unable to work^**4**^	372	*6*	32	*3*	0.42 (0.31 – 0.57)	
Pension/benefit is main source of income	1724	*27*	200	*18*	0.58 (0.47 – 0.71)	<0.001

1. Denominators may vary due to missing data.

2. Odds ratios (*OR*), 95% confidence intervals (*CI*) and P values using logistic regression using generalised estimating equations with robust standard errors.

3. *RRMA* Rural Remote and Metropolitan Areas classification [16,17].

4. Includes home duties, unpaid work and maternity leave.

Participant health characteristics are shown in Table 2. People who saw a chiropractor in the last 12 months were more likely to have a back problem, chronic back pain or sciatica (OR 2.90, 95% CI 2.53, 3.31). No important differences were seen between chiropractic users and non-users for other health variables, including health rating, arthritis, smoking or drinking rates, history of depression or anxiety, cardiovascular disorder, respiratory disorder and cancer. Chiropractic users, compared to non-users, were slightly less likely to use medications for certain health problems such as for blood pressure, cholesterol lowering and asthma (34% versus 38%; OR 0.83, 95% CI 0.72, 0.95) and were more likely to use complementary and alternative medication (35% versus 24%; OR 1.79, 95% CI 1.54, 2.07).

**Table 2:** Participant health characteristics (N = 7,519)

	**Visit to chiropractor in last 12 months**^**1**^					
	**No (N = 6385)**		**Yes (N = 1134)**			
**Participant health characteristics**	**Number**	***%***	**Number**	***%***	**OR (95% CI)**^**2**^	**P**
Health rate (SF 12) [18]:						
- Fair/poor	1056	*17*	165	*15*	REF	0.16
- Good/excellent	5253	*83*	956	*85*	1.16 (0.94 –1.43)	
Current smoker	1153	*18*	202	*18*	0.97 (0.82 – 1.15)	0.73
Hazardous drinking [19]	1033	*16*	197	*17*	1.06 (0.92 – 1.23)	0.42
Long term health problem limits daily activities	2021	*33*	345	*31*	0.94 (0.84 – 1.06)	0.29
Back problem in last 12 months^**3**^	1542	*24*	543	*48*	2.90 (2.53 – 3.31)	<0.001
Arthritis in last 12 months	1142	*18*	183	*16*	0.88 (0.74 – 1.06)	0.19
Cardiovascular disorder in last 12 months^**4**^	1543	*24*	241	*21*	0.84 (0.72 – 0.98)	0.03
Respiratory disorder in last 12 months^**5**^	948	*15*	190	*17*	1.15 (0.97 – 1.38)	0.12
Dermatitis in last 12 months	414	*7*	99	*9*	1.38 (1.12 – 1.71)	0.003
Diabetes in last 12 months	333	*5*	54	*5*	0.92 (0.68 – 1.24)	0.58
Cancer in last 12 months	162	*3*	26	*2*	0.91 (0.61 – 1.35)	0.63
Depression in last 12 months	1138	*18*	206	*18*	1.03 (0.86 – 1.23)	0.78
Depression and antidepressant use in last 12 months	591	*9*	94	*8*	0.89 (0.75 – 1.05)	0.15
Told by Dr you have depression	1821	*31*	337	*32*	1.07 (0.92 – 1.24)	0.38
Anxiety in last 12 months	1040	*16*	201	*18*	1.11 (0.96 – 1.29)	0.16
Told by doctor you have anxiety	1382	*25*	254	*26*	1.05 (0.90 – 1.23)	0.53
Afraid of partner	1003	*16*	184	*16*	1.02 (0.84 – 1.24)	0.81
Medication use in last 12 months:						
- Analgesics^**6**^	1677	*27*	279	*25*	0.92 (0.80 – 1.07)	0.28
- Medications for physical problems^**7**^	2412	*38*	381	*34*	0.83 (0.72 – 0.95)	0.009
- Depression medications	876	*14*	150	*13*	0.97 (0.79 – 1.19)	0.77
- Sedatives	466	*7*	77	*7*	0.95 (0.79 – 1.15)	0.61
- Other medications^**8**^	1476	*33*	256	*32*	0.96 (0.80 – 1.16)	0.70
- Complementary & alternative medication^**9**^	1488	*24*	395	*35*	1.79 (1.54 – 2.07)	<0.001

1. Denominators may vary due to missing data.

2. Odds ratios (*OR*), 95% confidence intervals (*CI*) and P values using logistic regression using generalised estimating equations with robust standard errors.

3. Includes back problem, chronic back pain or sciatica.

4. Includes stroke, hypertension, heart disease, lipid disorder.

5. Includes sinusitis, asthma and emphysema.

6. Includes painkillers and arthritis medication.

7. Includes blood pressure, cholesterol lowering & asthma medication.

8. Includes contraceptives and indigestion medication.

9. Includes vitamins, minerals and herbs.

People who reported seeing a chiropractor were more likely to report also visiting another type of complementary health practitioner (Adjusted OR 2.51, 95% CI 2.22, 2.84). They were also more likely to visit a physiotherapist (Adjusted OR 1.20, 95% CI 1.03, 1.39). They were not more or less likely to visit their GP more than 12 times in the last 12 months, nor more or less likely to have seen another “traditional” health practitioner (Table 3).

**Table 3:** Health service use in last 12 months of users of chiropractic compared to non-users

	**Non-chiropractic user**		**Chiropractic user**					
	**(N = 6385)**^**1**^		**(N = 1134)**^**1**^		**Unadjusted**		**Adjusted**^**2**^	
	**Number**	**%**	**Number**	**%**	**OR (95% CI)**^**3**^	**P**	**OR (95% CI)**^**3**^	**P**
**12 or more visits to GP**^**4**^	597	*9*	85	*8*	0.78 (0.61 - 0.99)	0.04	0.82 (0.65 - 1.04)	0.11
**One or more visits to traditional**^**5**^	4510	*71*	808	*71*	1.00 (0.90 - 1.10)	0.98	1.03 (0.92 - 1.14)	0.64
**One or more visits to complementary**^**6**^	974	*15*	351	*31*	2.53 (2.24 - 2.86)	<0.001	2.51 (2.22 - 2.84)	<0.001
**One or more visits to physiotherapist**	1276	*20*	246	*23*	1.19 (1.02 - 1.37)	0.03	1.20 (1.03 - 1.39)	0.02

1. Denominators may vary due to missing data.

2. Adjusted for age, gender, general practice location and health rate.

3. Odds ratios (*OR*), 95% confidence (*CI*) intervals and *P* values using logistic regression using generalised estimating equations with robust standard errors.

4. *GP* general practitioner.

5. Traditional includes: hospital doctor; specialist doctor; physiotherapist; psychologist; counsellor; psychiatrist; nurse; social worker; alcohol and drug worker; family therapist.

6. Complementary includes: naturopath; homeopath; acupuncturist; other natural therapist.

**Figure 1:**
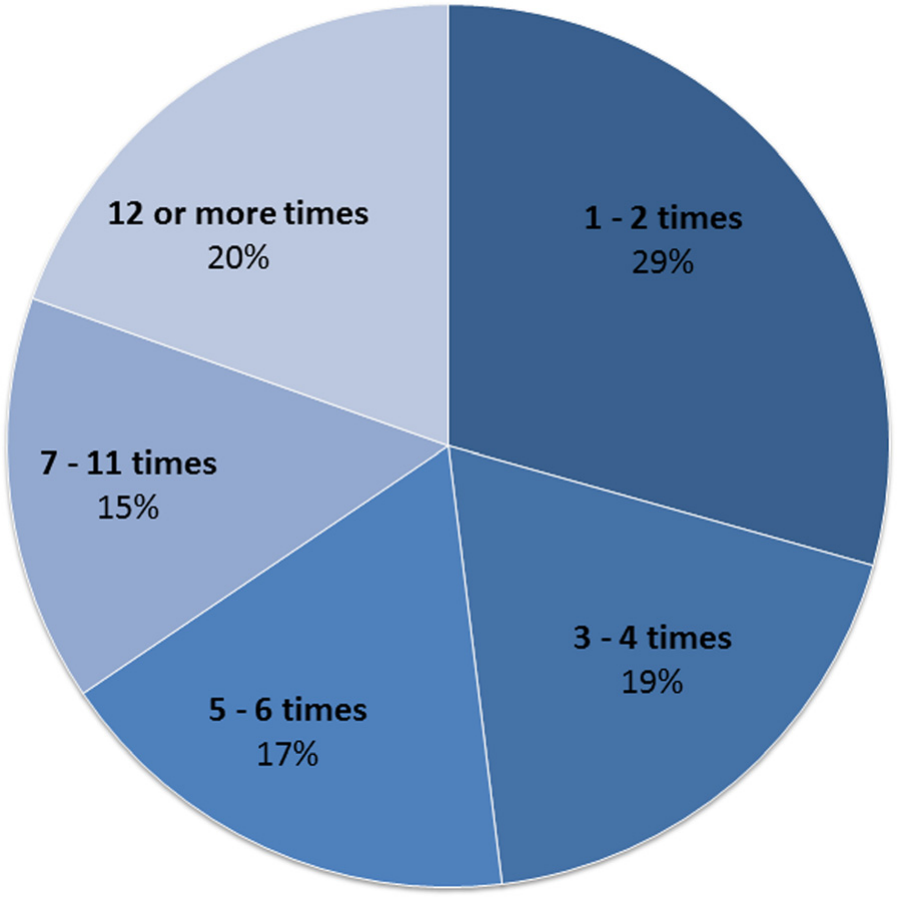
Number of visits to a chiropractor (N = 1,134).

**Figure 2:**
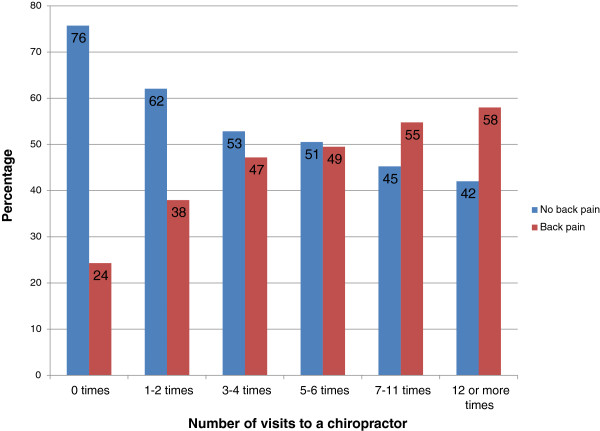
Number of visits to a chiropractor for people with and without back pain (back problem, chronic back pain or sciatica) (N = 7,477).

For those people who consulted a chiropractor, Figure 1 shows the number of visits over the last 12 months. About a third of people saw a chiropractor once or twice over the last 12 months, and one fifth saw a chiropractor 12 or more times. Figure 2 shows the number of visits to a chiropractor for people with and without back pain. The more often a person saw a chiropractor, the more likely it was that they had back pain; for example, 38% of people who saw a chiropractor 1 to 2 times in the last 12 months reported having back pain, whereas of those who saw a chiropractor 12 or more times in the last 12 months, 58% reported having back pain.

Table 4 shows participant health characteristics for people who visited a chiropractor and compares those who reported a back problem in the last 12 months to those who did not. People who had a back problem were more likely to report they had a long term health problem that limits their daily activities. They were also more likely to report a number of other chronic health conditions including arthritis, hypertension, chronic sinusitis, asthma, dermatitis, depression and anxiety, and were more likely to be a current smoker. They were not more or less likely to report a history of a stroke, heart disease, lipid disorder, emphysema, diabetes or cancer, nor more or less likely to report being afraid of their partner. Chiropractic users with a back problem were more likely to report taking medications, including analgesics, and complementary of alternative medications, and less likely to report taking medications for physical problems such as for blood pressure, cholesterol lowering and asthma. People with a back problem who reported seeing a chiropractor in the last 12 months were more likely to report having depression and to be taking antidepressant medication than those without a back problem.

**Table 4:** Participant health characteristics for those who visited a chiropractor comparing those who reported a back problem (back problem, chronic back pain or sciatica) in last 12 months to those who did not (N = 1,129)

	**Visited chiropractor and had self reported back problem in the last 12 months**^**1**^					
	**No (N = 586)**		**Yes (N = 543)**			
**Participant health characteristics**	**Number**	***%***	**Number**	***%***	**OR (95% CI)**^**2**^	**P**
Health rate (SF 12) [18]:						
- Fair/poor	82	*14*	83	*15*	REF	0.56
- Good/excellent	498	*86*	453	*85*	0.90 (0.63 - 1.29)	
Current smoker	87	*15*	114	*21*	1.53 (1.15 - 2.03)	0.003
Hazardous drinking [19]	99	*17*	98	*18*	1.09 (0.79 - 1.51)	0.61
Long term health problem limits daily activities	145	*25*	200	*38*	1.78 (1.33 - 2.37)	<0.001
Arthritis in last 12 months	60	*10*	123	*23*	2.57 (1.91 - 3.46)	<0.001
Cardiovascular disorder in last 12 months						
Stroke	2	*0*	4	*1*	2.14 (0.46 - 10.02)	0.34
Hypertension	71	*12*	89	*16*	1.43 (1.05 - 1.96)	0.02
Heart disease	8	*1*	19	*3*	2.59 (1.03 - 6.53)	0.04
Lipid disorder	57	*10*	63	*12*	1.21 (0.87 - 1.67)	0.26
Respiratory disorder in last 12 months						
Chronic sinusitis	26	*4*	53	*10*	2.33 (1.35 - 4.05)	0.003
Asthma	44	*8*	69	*13*	1.78 (1.21 - 2.61)	0.003
Emphysema	9	*2*	11	*2*	1.28 (0.47 - 3.52)	0.63
Dermatitis in last 12 months	38	*6*	61	*11*	1.81 (1.12 - 2.92)	0.02
Diabetes in last 12 months	28	*5*	26	*5*	1.00 (0.57 - 1.73)	0.99
Cancer in last 12 months	14	*2*	12	*2*	0.91 (0.49 - 1.68)	0.76
Depression in last 12 months	81	*14*	125	*23*	1.85 (1.30 - 2.64)	0.001
Depression and antidepressant use in last 12 months	32	*6*	62	*12*	2.23 (1.27 - 3.90)	0.005
Told by Dr you have depression	158	*30*	179	*36*	1.30 (0.99 - 1.71)	0.06
Anxiety in last 12 months	81	*14*	120	*22*	1.76 (1.22 - 2.55)	0.002
Told by doctor you have anxiety	127	*25*	126	*26*	1.08 (0.84 - 1.40)	0.52
Afraid of partner	97	*17*	87	*16*	0.95 (0.71 - 1.28)	0.73
Medication use in last 12 months:						
- Analgesics^**3**^	121	*21*	157	*29*	1.56 (1.17- 2.08)	0.003
- Medications for physical problems^**4**^	210	*36*	170	*32*	0.81 (0.64 - 1.03)	0.008
- Depression medications	71	*12*	79	*15*	1.24 (0.85 - 1.82)	0.23
- Sedatives	34	*6*	43	*8*	1.39 (0.81 - 2.38)	0.24
- Other medications^**5**^	119	*29*	135	*35*	1.36 (1.03 - 1.78)	0.03
- Complementary & alternative medication^**6**^	187	*32*	206	*38*	1.30 (1.02 - 1.64)	0.03

1. Denominators may vary due to missing data.

2. Odds ratios (*OR*), 95% confidence intervals (*CI*) and P values using logistic regression using generalised estimating equations with robust standard errors.

3. Includes painkillers & arthritis medication.

4. Includes blood pressure, cholesterol lowering & asthma medication.

5. Includes contraceptives and indigestion medication.

6. Includes vitamins, minerals and herbs.

## Discussion

This large cross-sectional study of patients of general practitioners suggests that 15% of people saw a chiropractor in the last 12 months and that chiropractors are the most commonly consulted complementary health profession. People who visit chiropractors are on the whole less disadvantaged (they are employed and they have completed secondary school education), and are more likely to be experiencing back problems. People who saw a chiropractor were also more likely to use complementary and alternative medication and to have visited another type of complementary or alternative health practitioner. People with back problems who visit a chiropractor are more likely to be depressed and to have some other chronic health problems.

These findings have important implications for chiropractors. If a consumer seeks their care for a back problem then that consumer is more likely to have a number of other chronic health conditions, including being a smoker, have arthritis, have hypertension, chronic sinusitis or asthma, dermatitis and depression or anxiety. Chiropractors should be aware of this and ensure that these people are assessed and appropriately managed for these other health conditions. In particular, people with an increasing number of chronic health problems are more likely to have depressive symptoms [20], so chiropractors should be particularly cognisant of this significant health problem.

The strength of this study is the large, representative sample of people living in the community. Even though the sample was drawn from general medical practice, four out of five Australians (82%) aged 15 years and over see their GP at least once per year [21].

The limitations of this study include that all data were collected by self-report. This may have led to recall bias in the respondent correctly identifying that the practitioner they consulted was a chiropractor, and also in remembering accurately the number of times they attended over the last 12 months. Also, because this study included analysis of secondary data, the study is limited by scope of questions, in that, the purpose of the questionnaire was not specifically designed to measure differences between people who did and didn’t see a chiropractor over the previous 12 months.

In a large study in the United States using National Health Survey data, 9% of respondents consulted a chiropractor in the last 12 months. Those that did were 2.4 times more likely to have low back pain [22]. Our results were similar to this with people visiting a chiropractor 2.9 times more likely to have low back pain. Other studies conducted in the United States have shown that people who visit chiropractors are more likely to be middle aged and to have high school as their highest level of education. Chiropractic patients were also more likely to have significantly worse health status than the general population sample [5]. We did not see these differences in our population.

## Conclusion

This large Australian cross-sectional study of general practice attendees suggests that chiropractors are the most commonly consulted complementary health profession. People who report seeing a chiropractor are more likely to be employed, have a back problem, have visited another type of complementary health practitioner and have a GP in a rural location. People who see a chiropractor and have a back problem are likely to have other chronic health conditions. Chiropractors should ensure they are aware of their patients’ health conditions other than musculoskeletal problems and should ensure these are appropriately managed.

## Competing interests

SF is an Associate Editor with Chiropractic & Manual Therapies and had no involvement in the editorial process for this paper. Otherwise, the authors declare that they have no competing interests.

## Authors’ contributions

SF and JG conceived and designed the study. KD and MC undertook the analysis. SF wrote the first draft of the manuscript. All authors contributed to revisions of the manuscript and read and approved the final version.
